# The prediction of hospital length of stay using unstructured data

**DOI:** 10.1186/s12911-021-01722-4

**Published:** 2021-12-18

**Authors:** Jan Chrusciel, François Girardon, Lucien Roquette, David Laplanche, Antoine Duclos, Stéphane Sanchez

**Affiliations:** 1 Pôle Territorial Santé Publique et Performance, Centre Hospitalier de Troyes, 101 Avenue Anatole France CS 10718, 10003 Troyes Cedex, France; 2 Research and Consulting, CODOC SAS, 75008 Paris, France; 3 Research on Healthcare Performance Lab, INSERM U1290 RESHAPE, Université Claude Bernard Lyon 1, Villeurbanne, France; 4 Health Data Department, Hospices Civils de Lyon, Lyon, France

**Keywords:** Emergency department, Length of stay, Data mining, Health services research

## Abstract

**Objective:**

This study aimed to assess the performance improvement for machine learning-based hospital length of stay (LOS) predictions when clinical signs written in text are accounted for and compared to the traditional approach of solely considering structured information such as age, gender and major ICD diagnosis.

**Methods:**

This study was an observational retrospective cohort study and analyzed patient stays admitted between 1 January to 24 September 2019. For each stay, a patient was admitted through the Emergency Department (ED) and stayed for more than two days in the subsequent service. LOS was predicted using two random forest models. The first included unstructured text extracted from electronic health records (EHRs). A word-embedding algorithm based on UMLS terminology with exact matching restricted to patient-centric affirmation sentences was used to assess the EHR data. The second model was primarily based on structured data in the form of diagnoses coded from the International Classification of Disease 10th Edition (ICD-10) and triage codes (CCMU/GEMSA classifications). Variables common to both models were: age, gender, zip/postal code, LOS in the ED, recent visit flag, assigned patient ward after the ED stay and short-term ED activity. Models were trained on 80% of data and performance was evaluated by accuracy on the remaining 20% test data.

**Results:**

The model using unstructured data had a 75.0% accuracy compared to 74.1% for the model containing structured data. The two models produced a similar prediction in 86.6% of cases. In a secondary analysis restricted to intensive care patients, the accuracy of both models was also similar (76.3% vs 75.0%).

**Conclusions:**

LOS prediction using unstructured data had similar accuracy to using structured data and can be considered of use to accurately model LOS.

**Supplementary Information:**

The online version contains supplementary material available at 10.1186/s12911-021-01722-4.

## Introduction

Length of stay (LOS) is a critical indicator for hospital management and has direct consequences on hospital costs and patient satisfaction. Moreover, LOS is correlated with disease severity and mortality [1]. When a patient is in the emergency department (ED), some predictors of hospital LOS are known before hospital admission. Studies have found patients at an ED are associated with a longer LOS [2–5] and patients who develop further complications in intensive care units (ICU) have a longer LOS beforehand at the ED [6]. For stroke patients, however, there is a significant inverse linear association between LOS at the ED and hospital LOS [7]. ED crowding and hospital occupancy at entry are predicted to have longer LOS [5, 8], however, there are other hospital characteristics that play a role in determining it [9, 10].

Patient characteristics also influence LOS, such as demographic characteristics and comorbidities which are often available at admission [11]. Depending on the medical specialty, physicians can predict LOS [12] although they tend to underestimate LOS in some cases such as patients with heart failure with LOS > 3 days [13, 14]. In psychiatry, patients have their own predictors such as a history of attempted suicide, which was negatively associated with LOS in a sample of 385 patients in Brazil [15]. These predictors are different according to age, where isolation plays a greater role for geriatric patients [16], but remains difficult to predict for psychiatric patients [17].

Although indicators can be compiled in bedside clinical scores like ALICE [18], statistical models can offer more flexibility for predictions of patient LOS. To date, logistic regression models have been used to predict discharge [19]. Cubist models have shown LOS prediction results [20] and tree-based models have presented improved performance and interpretability [21]. However, these models are usually run on structured data in tabular databases.

Most clinical data in electronic health records (EHRs) are presented in unstructured text form such as patient history narratives written by physicians. Although this data contains valuable information, it has rarely been used for automated predictions, particularly in the context of an ED. To date, these methods for knowledge extraction are not widely available in the medical field. Moreover, manual chart abstraction is time-consuming and expensive [22]. Automated information extraction from unstructured text can be simplified using controlled vocabularies [23, 24] like the Unified Medical Language System (UMLS). The objective of this study was to assess performance improvement for LOS prediction when accounting for clinical information written in text compared to the traditional approach of solely considering structured information.

## Methods

### Inclusion criteria

This retrospective study included patients admitted to the *Centre Hospitalier de Troyes,* a large French hospital situated in a rural region, between 1 January 2019 and 24 September 2019. Patient were included if they were admitted to the hospital through the ED and stayed more than two days for the subsequent hospital ward. Patients not admitted through the ED and patients with very short subsequent hospital stays (< 2 days) were excluded. The hospital under study had a Short Stay Emergency Ward. This unit has the capacity to host patients for several days, therefore, it is treated as any other medical ward. The time spent in the Short Stay Emergency Ward was accounted for in the total LOS.

### Data source

Patient stays and related features were selected and extracted all at once using the Dr Warehouse platform [25]. The information used for modelling was all information that was available to the ED staff at the time of the patient’s transfer to another ward of the hospital. This information included: i) personal information such as age, gender and zip/postal code, ii) context information such as entry date, LOS at the ED, triage (CCMU and GEMSA) codes, iii) ICD-10 primary diagnosis code and iv) unstructured information such as the UMLS concepts extracted from the text documents uploaded during the stay at the ED.

### Ethical and regulatory considerations

The study was declared to the French registry of studies using healthcare data (N° F20210719114017). The study was conducted in compliance with French MR004 regulation (*Commission Nationale Informatique et Libertés*). Since the study was retrospective and was based on pseudonymized data and purely observational, it was exempt from Institutional Review Board approval according to the French Public Health Code (L1121-1, Law number 2012-300, 5 March 2012).

### UMLS concept extraction

UMLS is a meta-thesaurus and ontology of medical concepts created by the National Library of Medicine (USA) covering a broad range of concepts from anatomy to physiology and medical semiology. It includes vocabularies from SNOMED CT, RxNorm, LOINC, MeSH, CPT, ICD-10-CM, MedDRA, the Human Phenotype Ontology and other sources. We used the UMLS detection module of the Dr Warehouse platform [26] to extract UMLS concepts from free text in the EHRs at the ED. The main computation steps used for the extraction were [27]: i) to split the free-text into a collection of sub-text (sentences, or propositions) using punctuation and text structure, ii) to classify each sub-text within the following categories: “patient related—affirmation”, “patient related—negation”, “family related—affirmation”, “family related—negation”, where affirmation stood also for neutral context, and iii) for each sub-text labelled as “patient related—affirmation” to find the most precise concepts that exactly match concepts of the UMLS thesaurus within this sub-text. The UMLS tree-structure was leveraged to reach the most precise concept that is a concept leaf of the tree.

To address the issue of high prevalence of some concepts, a variation of the relevance frequency concept [28] was used to filter out non-relevant concepts. For each extracted concept we computed the *srf* (symmetric relevance frequency) score as follows:

$$srf = \log_{2} (2 + \max \, \left( {{\text{a/}}\max \left( {1,{\text{c}}} \right),\;{\text{c/}}\max \left( {1,{\text{a}}} \right)} \right)$$

where a is the number of long stays (≥ 7 days) in which the concept is found and c is the number of short stays (< 7 days) in which the concept is found. All concepts for which the prevalence of one class over the other was under the 45% threshold, meaning *srf* ≤ log_2_(2 + 55/45) = 1.688, were marked as non-relevant.

### ICD-10 diagnostic codes

Numerous diagnoses have a low number of occurrences. To tackle this issue, the hierarchical structure of the ICD-10 diagnosis code was leveraged in our study. For each diagnosis code, if the number of occurrences was lower than five, we replaced it with its parent in the hierarchy, stopping at the three characters level (such as C00) to avoid losing too much information. For example, if M6289 appeared in less than five stays then it was replaced by M628. If this code still appeared in less than five stays, it was then replaced by M62.

### CCMU classification code

The CCMU classification code consists of either “P” if the patient presents psychiatric symptoms, “D” if the patient is deceased on arrival or a number between 1 and 5 depicting the patient’s condition (1: stable and 5: vital prognosis engaged). The numbers were left unchanged; however, the letters had to be replaced by numerical values. The letter D was replaced by the number 6 and the letter P was replaced with the number 0.

### Added features

To improve model performance, several features were built using the available data. Firstly, the “recent prior visit” feature was built by looking at previous admissions in the ED for each patient. The “recent prior visit” value was defined as 1 if a patient had already been admitted at least once in the seven days prior to the given stay and 0 otherwise. We obtained a total of 656 (13%) stays with the flag set to 1 out of the total stays.

Another added feature was the short-term ED activity index since ED crowding has been shown to help predict patients’ overall LOS [29]. Although there were other determinants of crowding (for example, the number of beds available outside of the ED), crowding was expected to occur with increased frequency when the number of incoming patients was unusually high. For each ED admission, we counted the number of admissions that occurred during the seven previous days, and then: i) if the count was under the 1st decile of prior-admission counts then the index was set to 0, ii) if the count was over the 9th decile of prior admission counts then the index was set to 1 and iii) if the count was between the two values, then the index was linearly interpolated. This indicator also captures seasonal effects, being low in periods of the year during which patients are less likely to come to the ED of the hospital under study.

To produce a fair comparison, the following three sets of features were chosen: i) features common to both sets including age, gender, zip/postal code, LOS at the ED, recent visit flag, short-term ED activity index, hospital service after ED stay, ii) “structured data only” set including CCMU, GEMSA & ICD-10 codes, iii) “unstructured data included” set: UMLS concepts.

Personal information of patients and the context were kept for both featured sets. In the first set, the structured diagnosis information was added, whereas in the second set, only the clinical data directly extracted from the text notes was added. The variables used for the structured data model and for the unstructured model are summarized in Additional file 1: Table S5. Additional information on how the data was encoded for the Random Forest Model can be found in Additional file 2: Appendix 2. Although the same kind of model was used for both featured sets, each set’s model had its own set of hyper-parameters. Both sets of hyper-parameters were computed independently to optimize performance in each case. This allowed us to compare the best achievable model for both set of features.

### Model

To alleviate the problems inherent to the modelling of long-tail distributions such as LOS, we decided to reduce the inference scenario to a binary classification defined by the ad-hoc threshold of seven days. This threshold represented the median LOS for our dataset ensuring balanced classes in the classification outcomes. A random forest model was used to predict the “long stay” and “short stay” classes of the LOS variable. Long stays were defined as lasting longer than the LOS median of six days. One motivation for the choice of a random forest model was the distribution of the classes shown in Additional file 1: Figure S1 (Appendix 1). Indeed, the decision region shapes needed to correctly encompass each class were too complex for linear or kernel-based models. A tree-based model, however, could sufficiently produce complex decision regions. Moreover, random forests have unique properties like the reduction of overfitting by averaging multiple decision trees [30].

It is worth noting that the ICD-10 diagnosis codes and UMLS Concepts are categorical features, meaning that to be used by the machine learning models, they had to be encoded using One-Hot Encoding (each category value is converted into a new column and assigned a 1 or 0 (notation for true/false) value to the column. In this study, there were 969 UMLS concepts and each stay had 17.23 associated UMLS concepts on average. Whereas there were 222 ICD10 diagnostic codes with 1 per stay showing numerous co-occurrences of UMLS concepts. This translated to a high multi-collinearity between concepts, which is a problem for linear and kernel-based models.

### Model hyperparameter tuning

The method used to choose the optimal set of hyperparameters for each feature set were described. This method consisted of using a random search to go through hyperparameter combinations (within previously defined bounds) and evaluate the model’s performance with each one using cross-validation. The set of hyperparameters used for the final model was the set that produced the best model accuracy. The list of all possible values for each hyperparameter can be found in Additional file 1: Table S1 (Appendix 1).

The key hyperparameters used were: i) n_estimators: This parameter determined the number of decision trees that constituted our forest. Additional trees, up to a certain point, improve model performance, ii) min_samples_split: This controlled the minimum number of samples required for a split to be able to happen on a node. Too high values led to under fitting as trees were not able to split enough times to achieve high-purity leaves. *Note:* We placed the lower bound to 5 to allow splitting even when considering infrequent diagnosis codes and iii) min_samples_leaf. Similar to ii), this parameter set a minimum number of samples required for a leaf node after splitting. The minimum value for this one was set low enough to account for the very infrequent diagnosis codes.

With the hyperparameter ranges of values defined, a random search was used to go through combinations of hyperparameters. Through this method, each iteration produced a different, randomly-chosen combination of hyperparameter values. Each combination was then evaluated using cross validation. The process of evaluating a model through cross validation started with partitioning the dataset into several “folds”, in other words subsets of equal size. For every such fold, the model was fitted on the union of all the other folds and its score was evaluated on the given fold (which was left out in the model fitting). The mean of the scores obtained in that manner constituted the score of the set of hyperparameters.

### Primary outcome

The primary outcome of this study was accuracy = TP + TN/(P + N) as the score, where TP is True Positives, TN is True Negatives, P is Positives and N is Negatives with long stays being considered positives. We used three folds and also recorded other indicators: i) sensitivity: proportion of actual long stays (≥ 7 days) predicted as such, ii) specificity: proportion of actual short stays (< 7 days) predicted as such, iii) precision: proportion of correct predictions among predicted long stays and iv) accuracy: proportion of correct predictions.

Both models were fitted on a subset made of 80% of the dataset (training set) and evaluated on the rest (test set). Furthermore, both models were fitted on the exact same training set and evaluated on the exact same test set. Each model used its own set of hyperparameter values obtained using the method described earlier with the same number of iterations on the random search.

## Results

### Patient characteristics

In total, 5,006 patients were included in the study. Patient characteristics are presented in Table 1. The admission rate in the ED of the hospital under study was 28.7% in 2019. The types of stays registered in the ED of this hospital were very diverse: even the most prevalent diagnoses had a relatively low number of occurrences. This was not the case for the UMLS concepts, with the top two most frequent UMLS concepts being present in more than 91% of all stays (Additional file 1: Figure S2, Appendix 1).

**Table 1:** Patient characteristics for the study to assess the prediction of hospital length of stay (LOS) using unstructured data at the emergency department (ED)

Characteristic	Total
n	5,006
Age—mean ± SD	64.3 ± 26.3
Age category—n (%)	
< 18	494 (9.9)
Age ≥ 18	4512 (90.1)
Gender—n (%)	
Male	2,333 (46.6)
Female	2,673 (53.4)
Emergency LOS (hours)—Median (Q1–Q3)	7.2 (4.8–9.6)
Total (ED + hospital) LOS (days)—Median (Q1–Q3)	6.1 (3.7–11.0)
Intensive care patients—n (%)	378 (7.6)
Most frequent diagnoses—n (%)	
Pneumonia (J189)	212 (4.2)
Altered general health (R53 + 0)	188 (3.8)
Shortness of breath (R060)	174 (3.5)
Abdominal pain (R104)	122 (2.4)
Femoral bone fracture (S7200)	121 (2.4)
Most frequent concepts—n (%)	
Pain	4,921 (98.3)
Blood pressure	4,568 (91.3)
Capillary	3,521 (70.3)
Abdomen	2,155 (43.0)
Face	2,046 (40.9)
Type of hospital stay, n (%)	
Pulmonology	871 (17.4)
Digestive system	761 (15.2)
Cardiovascular medicine (except cardiovascular catheterization)	503 (10.0)
Trauma and orthopaedics	467 (9.3)
Diseases of the nervous system (including stroke)	465 (9.3)
Urology, nephrology	332 (6.6)
Rheumatology	313 (6.3)
Endocrinology	195 (3.9)
Hematology	193 (3.9)
Diagnostic or therapeutic catheterization	161 (3.2)
Dermatology	134 (2.7)
ENT, stomatology	128 (2.6)
Toxicology, alcohol-related disease	122 (2.4)
Psychiatry	107 (2.1)
Multidisciplinary stays and known disease follow-up	89 (1.8)
Obstetrics	51 (1.0)
Infectiology	44 (0.9)
Gynecology	38 (0.8)
Other (chronic pain, ophtalmology, complex trauma, burn injury)	32 (0.6)

### Model results and performance

Overall, model performance of the two models (unstructured data vs structured data) were similar. The set of hyperparameter values chosen for each model are presented in Additional file 1: Table S2. Examples using other parameters are presented in Additional file 1: Figure S3 (Appendix 1). These values were produced using 50 iterations on the random search and 3 folds in the cross validation.

Table 2 shows the performance of each model. Including the clinical data extracted from text notes produced in the ED led to a small increase in predictive performance, from 74.1% to 75.0% (with an F1-score change from 75.7% to 76.4%). The two models concurred in 86.6% of predictions. The number of records for which the two model predictions differ or concur is highlighted in Additional file 1: Table S3. As shown in Additional file 1: Table S4 (Appendix 1), there was a distinction between the characteristics of EHRs which the models produced the same prediction and those for which the predictions were different.

**Table 2:** Model performance for the “structured-data only” and “unstructured-data added” feature sets

	Structured	Unstructured	Difference (pts)	All features
Recall	77.3%	77.1%	− 0.19	76.6%
Specificity	70.4%	72.7%	2.31	71.1%
Precision	74.2%	75.7%	1.48	74.4%
Accuracy	74.1%	75.0%	1.0	75.0%
F1 Score	75.7%	76.4%	0.68	75.5%

**Fig. 1:**
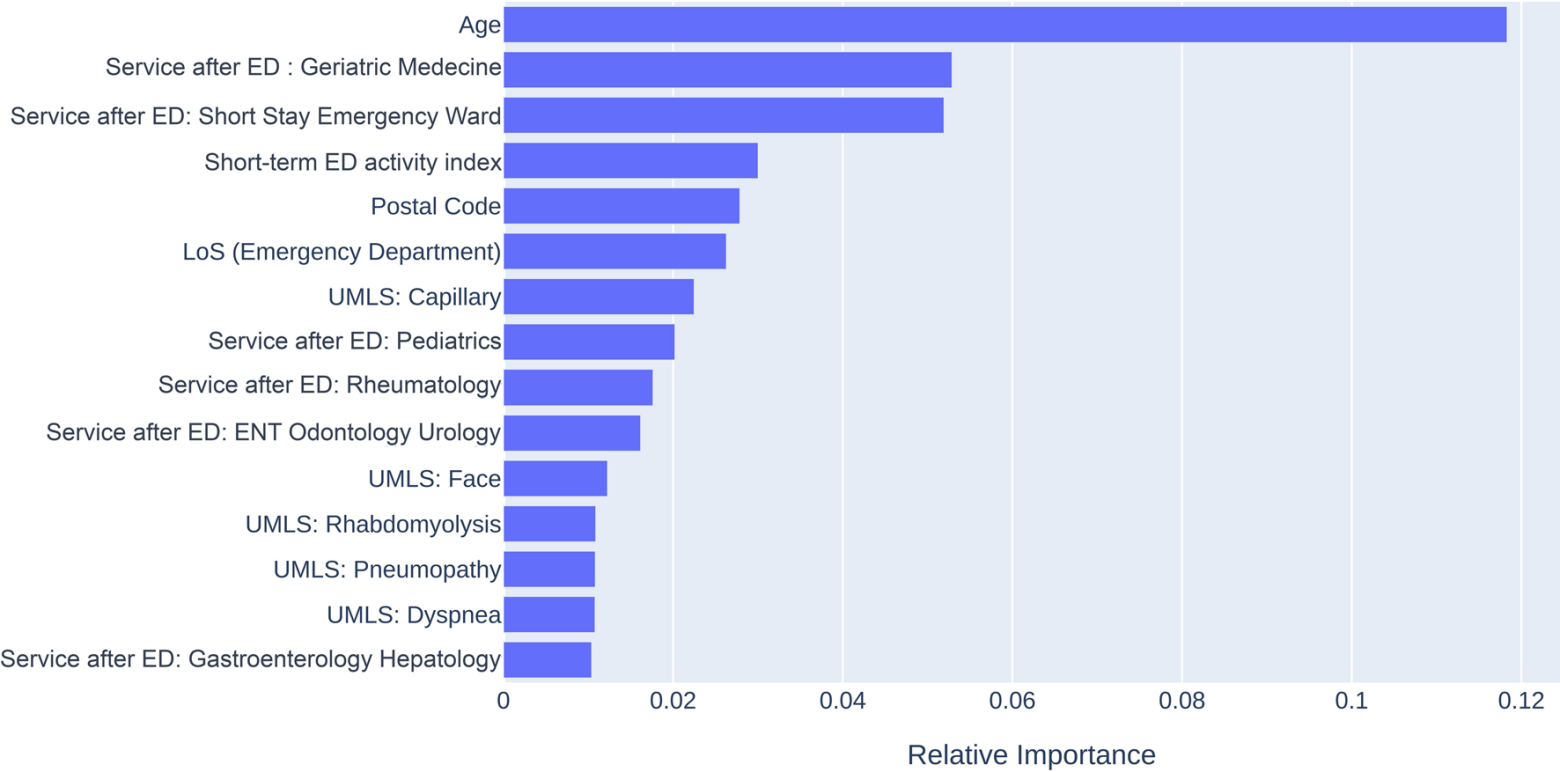
Feature importance for the unstructured data model to predict hospital length of stay (LOS)

**Fig. 2:**
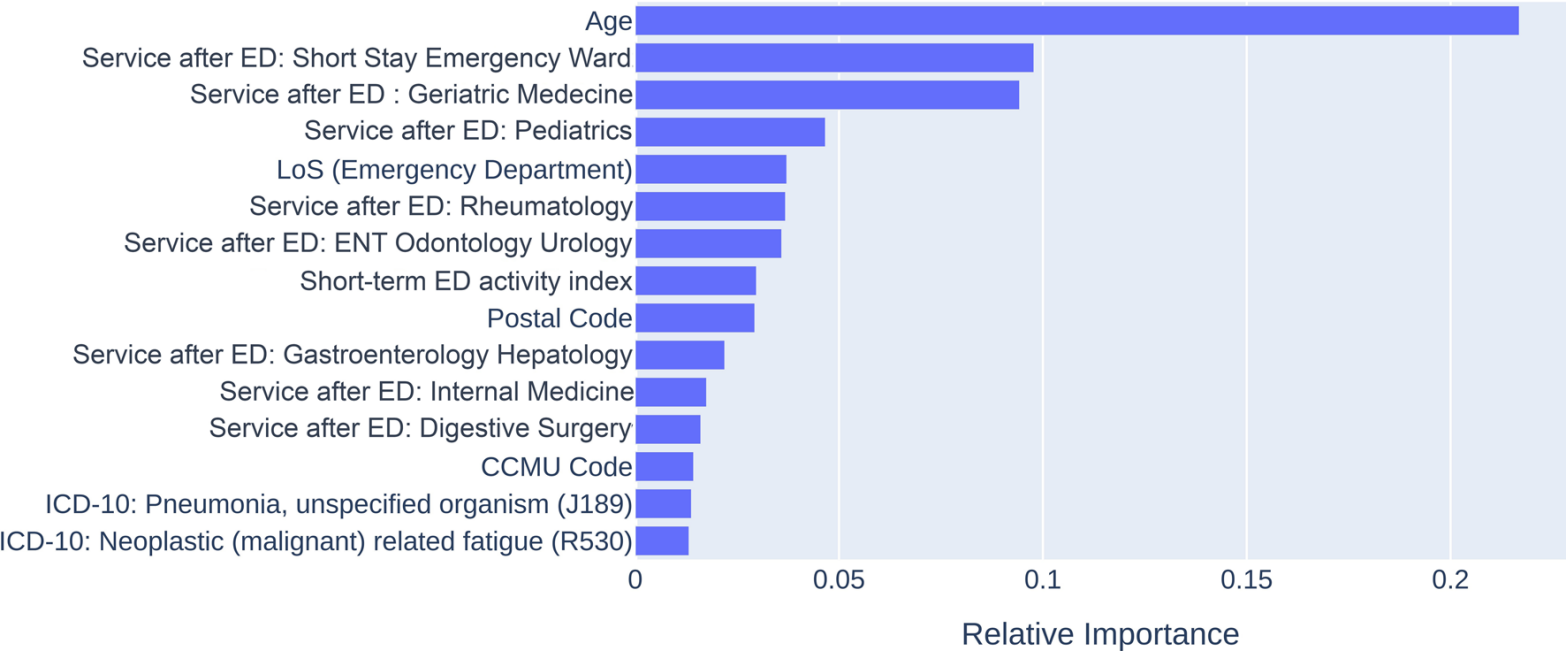
Feature importance for the structured data model to predict hospital length of stay (LOS)

Figures 1 and 2 present the relative importance of the features for both the unstructured data and structured data models. Age was the most determining factor in predicting LOS. Another important feature was the short-term ED activity index. Regarding UMLS concepts, the presence of “capillary” in the ED health record was associated with the presence of a standardized vital parameters surveillance chart (which included the measure of capillary glycaemia) and in turn influenced the probability of a long stay. A secondary analysis measured the performance of the two models for LOS prediction of ICU patients. The training set of 378 ICU patients was used to train the model, which was tested on the remaining 76 patients. In this analysis, the unstructured data model achieved better accuracy than the structured data model (76.3% versus 75.0%) (Table 3). Feature importance for the two models limited to intensive care stays are presented in Additional file 1: Figure S4 and Figure S5 (Appendix 1).

**Table 3:** Model performance of structured and unstructured data to predict hospital length of stay (LOS) when trained on intensive care unit stays

	Structured data	Unstructured data	Difference (points)	All features
Recall	77.6%	75.9%	− 1.72	84.5%
Specificity	66.7%	77.8%	11.11	72.2%
Precision	88.2%	91.7%	3.43	90.7%
Accuracy	75.0%	76.3%	1.32	76.3%
F1 score	82.6%	83.1%	0.49	87.5%

## Discussion

This study showed that UMLS-based one-hot vector word-embedding within an affirmative patient-centric context from EHRs is an effective way to predict LOS at an ED when using machine learning (random forests). The accuracy of the model using unstructured data was similar to the accuracy obtained using structured data. Therefore, this shows that unstructured data should also be considered in its use to obviate the need for resource-intensive data abstracting conducted by humans. The accuracy remained adequate despite the exclusion of very short stays, which could be easier to predict in some cases. Even though the increase in accuracy when unstructured data was used was small, it should be noted that this data set did not contain any of the structured diagnostic information (ICD-10 codes) of the structured model.

Moreover, the unstructured data model performed similarly or better than the structured data model for intensive care stays. ICU patients often have a highly standardized management that involves numerous medical examinations and procedures. Data pertaining to these elements are often recorded in the patient’s EHR and thus contains relevant information for the determination of LOS.

We used random forest models to predict LOS at the ED since this model is well suited for treating data with complex interactions between variables and other non-linear effects. Models based on deep neural networks are another option that could be explored in further studies. Such models have been used to predict admission or discharge of ED patients with better F1-score performance than logistic regression (31), although the obtained F1-score of 0.674 seems low compared to our findings.

In the literature, Roquette et al. used deep neural networks with their *text2num* embedding method (in the context of pediatric ED prediction admission using unstructured text data) and obtained results very similar to ours with a recall and specificity of approximately 80% and a 1.8 point increase in the Area Under the Curve after adding unstructured data [32]. However, in this design it could have been possible that the endpoint was in some cases directly encoded in the training data in the form of emergency physician recommendations regarding admission or discharge.

In another study by Zhang et al. [33] unstructured text improved predictions only when used in conjunction with structured data. Joseph [34] used free text to identify critically ill patients, enabling an increase in the Area Under Curve compared to structured only data models (with an AUC of 0.851 [95% CI: 0.849 to 0.852]). Choi [35] used random forests and gradient boosting to predict ED triage status and enriched their model with free text nursing triage notes. Both models had comparable performance with an Area Under Curve of 0.92 and in each case the best performance was achieved after the addition of text data. Other studies processed unstructured text data in an automated manner to make healthcare predictions regarding mortality [36], disease association patterns [37, 38], or risk areas in medication administration [39].

Regarding accuracy, the accuracy of predictions depends on the quantity and relevance of variables included. At our hospital, socio-economic status is not routinely extracted in health records and were not recorded in this study. Further research into unstructured text-mining methods could extract concepts relevant to this characteristic type. The use of unstructured data in predictive models based on generic, automated and replicable extraction pipelines is of primary interest for scalability purposes of such models on EHR systems, though this desirable scalability property comes with an additional technicality cost.

Two key limitations to this study were the high dimensionality of data and the signal-to-noise ratio within extracted semantic concepts. The first limitation was a common issue and could be tackled with regularization methods such as L1-penality (such as LASSO). The second was intrinsically linked to the retained extraction pipeline. In this study, we leveraged the well-established UMLS terminological system and extraction pipeline embedded in Dr Warehouse [25–27]. The UMLS meta-thesaurus is the richest collection of terminologies available with over 4.4 million medical concepts. This choice may guarantee cross-applications forecasting capabilities, however the signal to noise ratio may not be fully optimized for the specific LOS prediction problem. This observation motivates our pre-processing method using the symmetric relevance score. More sophisticated word-embedding methods could improve the performance of machine learning algorithms by using contextual information, including diagnostic hypotheses, patient comorbidity and patient history, to filter only the most relevant concepts and relations among them.

Although the sample size in our study was adequate, the addition of other centers could have enhanced the generalizability of our results. Single-center studies, however, provide locally actionable insights that could be used to inform quality improvement interventions and other hospitals could train similar models on their own data which could provide results tailored to their needs.

While LOS was considered as a categorical variable to maximise the power of the model, prediction of the LOS as a continuous variable could be a target for future studies. Only the presence of UMLS concepts are considered, and not the context surrounding these concepts, which might warrant investigation in future research.

## Conclusions

This study shows that unstructured data (free text) can be used to predict LOS with acceptable predictive performance. The performance was similar to the performance of the model using structured data. Structured data, however, may have the drawback of being more time-consuming to extract. In many applications, unstructured text data contains valuable insights that are yet to be explored. As the methods to automatically extract knowledge evolve, they will undoubtedly give more accurate predictions. Modules to extract specific information like the primary complaint [40] or presence of pain [41] are currently being developed and could be combined or added to already existing software [42–44]. Future research needs to determine how these methods can ultimately improve healthcare outcomes while complying with privacy laws and maintaining high ethical standards.

## Supplementary Information

**Additional file 1**. Supplementary figures and tables related to model specifications, modeling parameters and modeling results.

**Additional file 2**. Data formats used for modeling.

## Data Availability

All data material can be accessed upon request to the last author Dr Stéphane Sanchez at the following email address stephane.sanchez@hcs-sante.fr.
