# Supplementary material for: The prediction of hospital length of stay using unstructured data
Source: BMC Med Inform Decis Mak. 2021 Dec 18;21:351. doi: 10.1186/s12911-021-01722-4 (PMC8684269; doi:10.1186/s12911-021-01722-4)
Supplement: Supplementary file 1 — Additional file 1. Supplementary figures and tables related to model specifications, modeling parameters and modeling results. [file 12911_2021_1722_MOESM1_ESM.docx]

**Appendix 1**


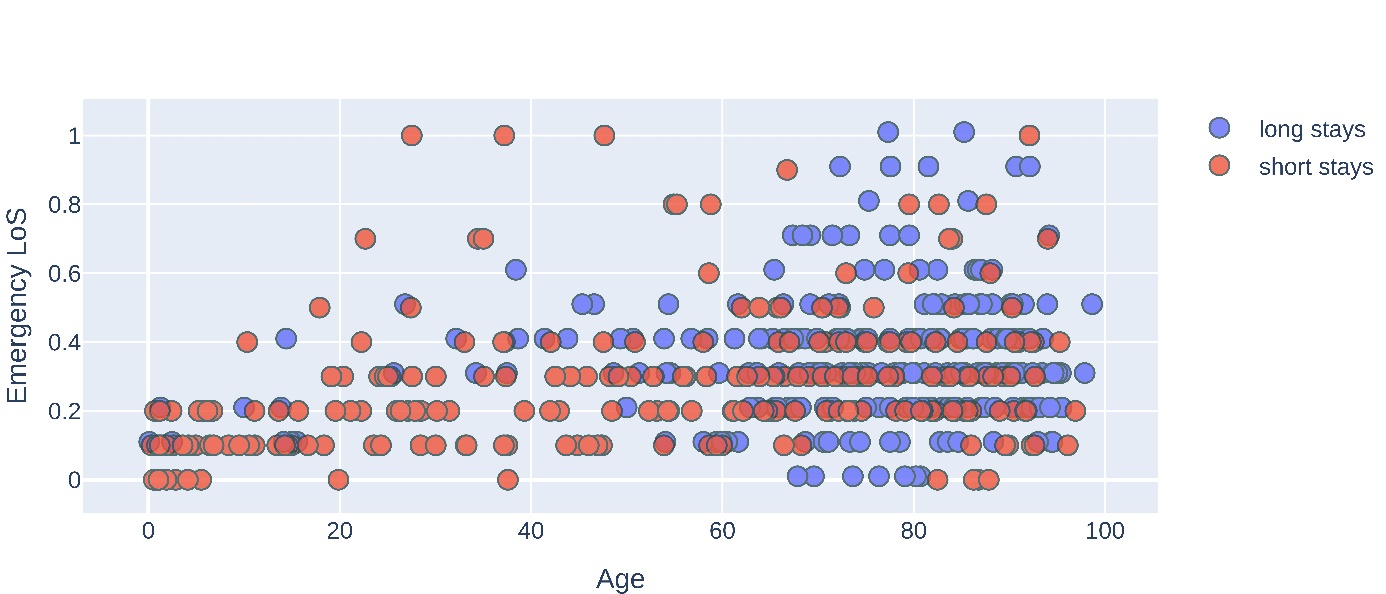


**Figure S1.** Length of stay (LOS) class distribution in the plane defined by (age, ED LoS), Sample of 500

**Table S1.** List of possible values of Random Forest Hyperparameters

| Hyperparameter | Possible Values |
| --- | --- |
| Number of estimators | [100, 153, 206, 260, 313, 366, 420, 473, 526, 580, 633, 686, 740, 793, 846, 900] |
| Maximum features | [auto, sqrt] |
| Maximum depth | [10, 155, 300, 445, 590] |
| Minimum samples split | [5, 10, 15, 20, 30, 40, 50] |
| Minimum samples leaf | [2, 3, 4, 5, 10, 15, 20] |
| Bootstrap | [True, False] |


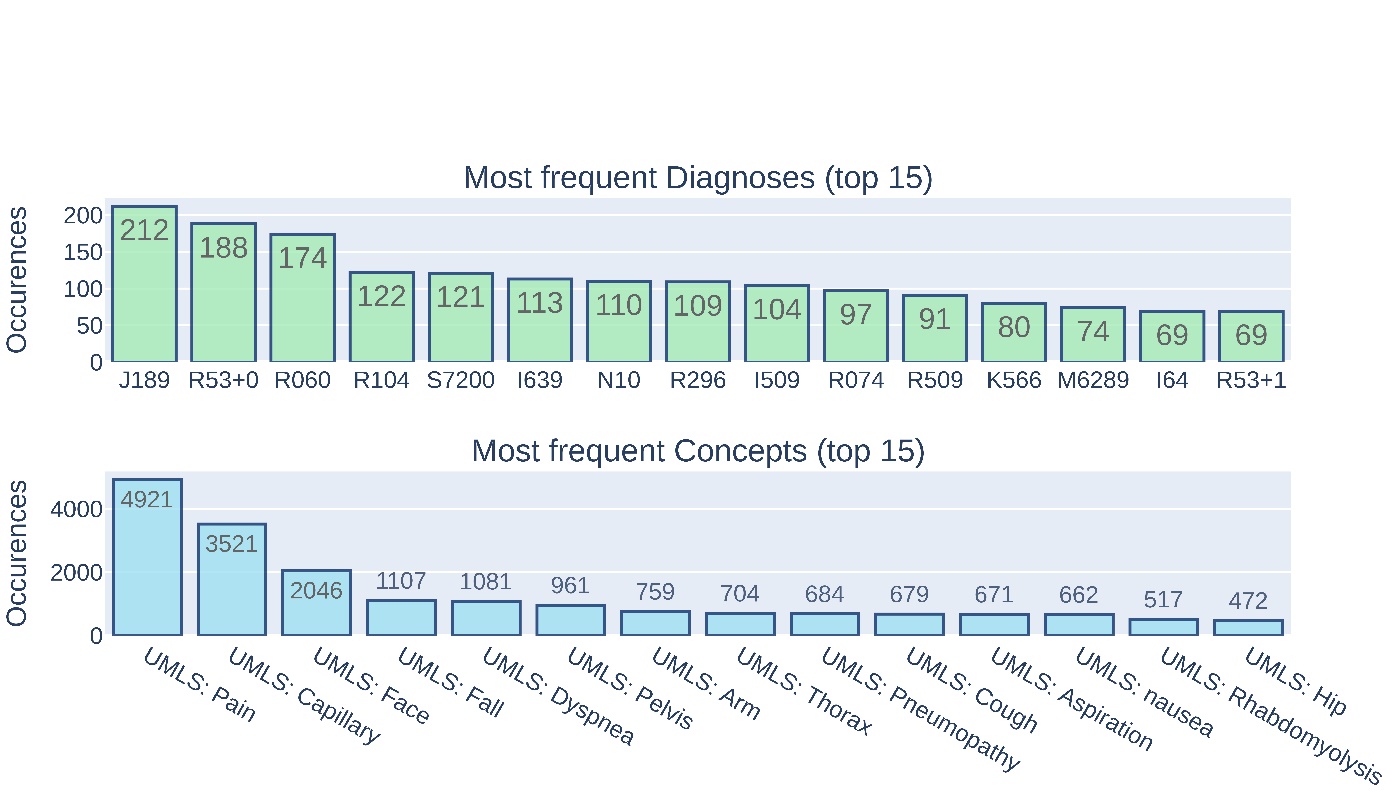


**Figure S2.** Most frequent values of ICD-10 Diagnosis and UMLS Concepts features of the dataset to predict hospital length of stay (LOS)

**Table S2.** Hyperparameter values chosen for the “structured-data only” and “unstructured-data added” feature set models

| Hyperparameter | Structured data only | Unstructured data |
| --- | --- | --- |
| Number of estimators | 420 | 473 |
| Min samples split | 20 | 5 |
| Min samples leaf | 3 | 4 |
| Max features | sqrt | sqrt |
| Max depth | 445 | 590 |
| Criterion | Gini | Entropy |
| Bootstrap | False | True |


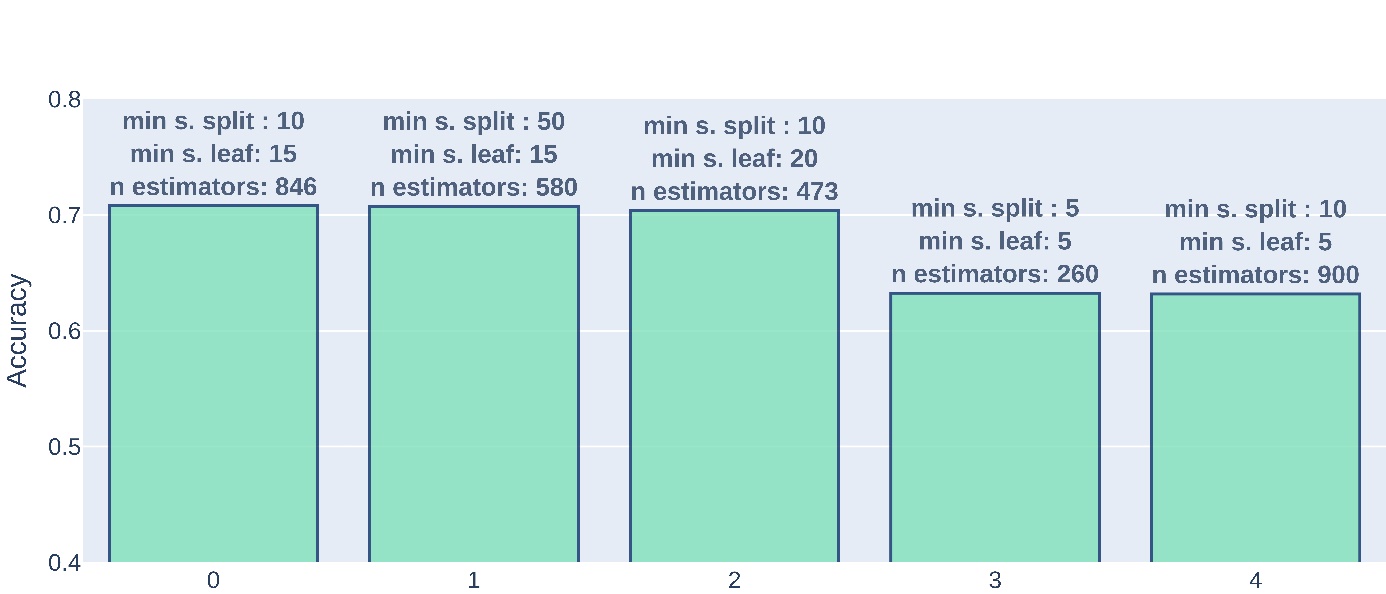


**Figure S3.** Hyperparameter accuracy scores sample to predict hospital length of stay (LOS)

**Table S3.** Concurrence of the model using unstructured data with the model using structured data to assess the prediction of hospital length of stay

| Actual LOS days) | Structured Model Prediction (days) | Unstructured Model prediction (days) | | Total |
| --- | --- | --- | --- | --- |
|  |  | [2, 7[ | [7, 85[ |  |
| [2, 7[ | [2, 7[ | 305 | 31 | 336 (TN) |
|  | [7, 85[ | 42 | 99 | 141 (FP) |
|  | Total | 347 (TN) | 130 (FP) |  |
| [7, 85[ | [2, 7[ | 89 | 30 | 119 (FN) |
|  | [7, 85[ | 31 | 375 | 406 (TP) |
|  | Total | 120 (FN) | 405 (TP) |  |

TN; true negatives, FN; false negatives, TP; true positives, TN; true negatives

**Table S4.** Differences in predictions between unstructured and structured data to assess hospital length of stay (LOS)

| Patient characteristic | Same prediction for models 1 and 2 | Different prediction |
| --- | --- | --- |
| Age (mean-median) | 63.7 - 71.7 | 71.5 - 73.4 |
| LOS at the ED (mean-median) | 0.33 - 0.3 | 0.31 - 0.3 |
| LOS (mean-median) | 9.69 - 7.0 | 8.46 - 6.0 |
| Gender, male (%) | 50.0% | 41.0% |
| Recent visit flag (%) | 13.0% | 14.0% |
| ZIP/postal code (top 5) | 10000  10120  10150  10600  10800 | 10000  10120  10200  10600  10140 |
| Post-ED Service (top 5) | Pediatrics; Geriatric Medicine; Cardiology; Neurology; Orthopedic surgery | Orthopedic surgery; Gastrointestinal surgery; Neurology; Cardiology; Short Stay Emergency Ward |
| ICD-10 Diagnosis (top 5) | Pneumonia, unspecified organism (J189),  Fainting and tiredness (R53+0),  Dyspnea (R060),  Abdominal pain, unspecified (R104),  Acute tubulo-interstitial nephritis (N10) | Fracture of unspecified part of neck of femur (S7200),  Unspecified chest pain (R074),  Unspecified cerebral infarction (I639),  Unspecified fracture of unspecified femur (S7290),  Unspecified stroke as hemorrhage or infarction (I64) |
| UMLS Concept (top 5) | Capillary  Face  Fall  Dyspnea  Fever | Capillary  Face  Waist  Fall  Hip |

LOS: length of stay; ED: emergency department; ICD-10: International Classification of Diseases, 10^th^ Edition; UMLS: Unified Medical Language System

**Table S5.** Summary of the features used by each model and the features used by both models. The categorical features (Service after ED, ICD-10 diagnosis and UMLS concepts found) are represented as they are used by the model, in other words in their indicator form.

| Structured Model Features | Unstructured Model Features |
| --- | --- |
| CCMU Code | UMLS: Cutaneous abscess |
| GEMSA Code | UMLS: Respiratory Alkalosis |
| ICD-10: A090 | .... |
| ICD-10: A099 | UMLS : Pulmonary Tuberculosis |
| .... |  |
| ICD-10: Z60 |  |
| Common features | |
| Age | |
| Gender | |
| Postal Code | |
| LoS in the ED | |
| Recent prior visit | |
| Short-term ED activity index | |
| Service after ED: Short Stay Emergency Ward | |
| Service after ED: Cardiology | |
| Service after ED: Digestive Surgery | |
| .... | |
| Service after ED: Rheumatology | |

Los: Length of stay; ED: Emergency department


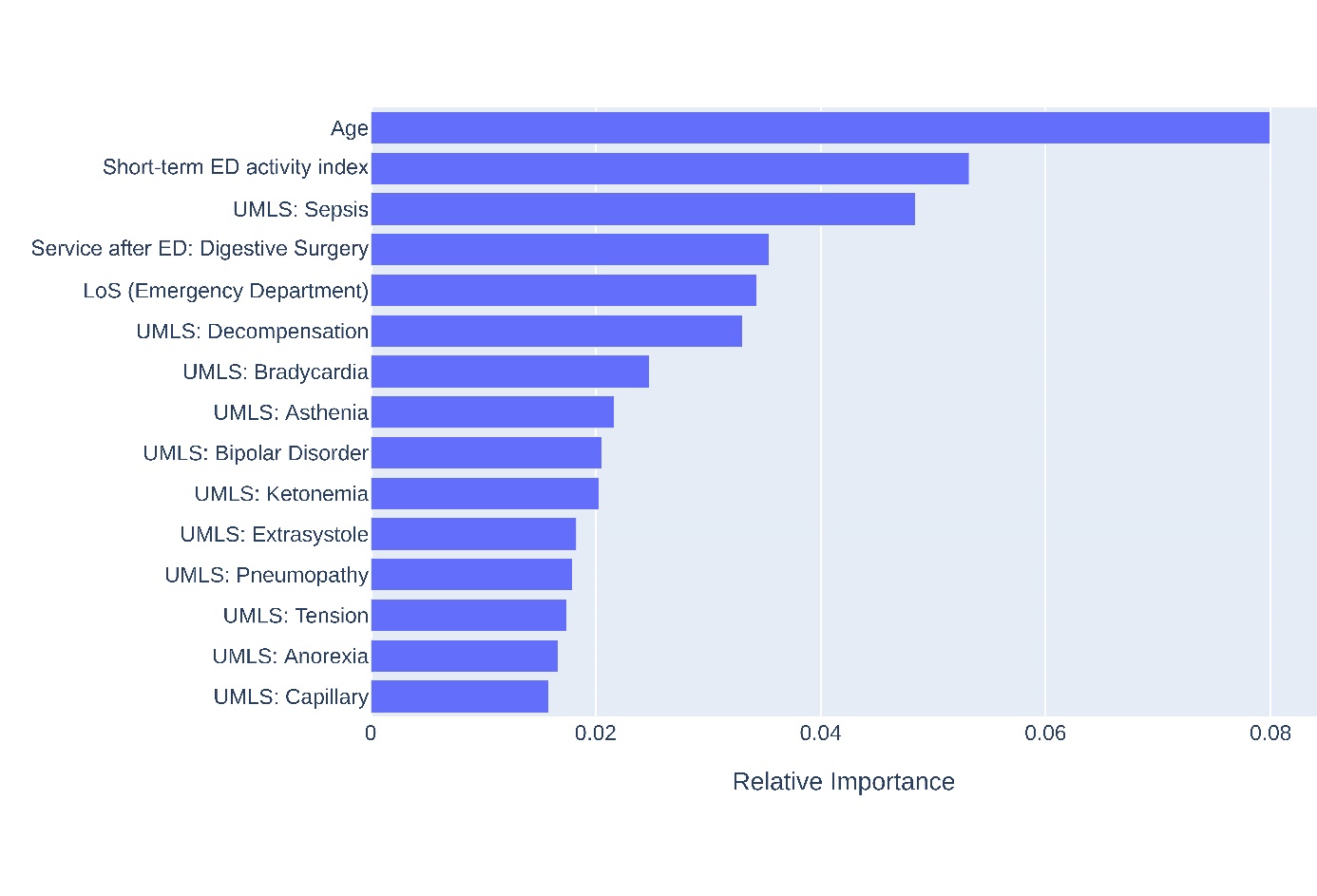


**Figure S4.** Feature importance for the unstructured data model in intensive care unit (ICU) patients to predict hospital length of stay (LOS)

**
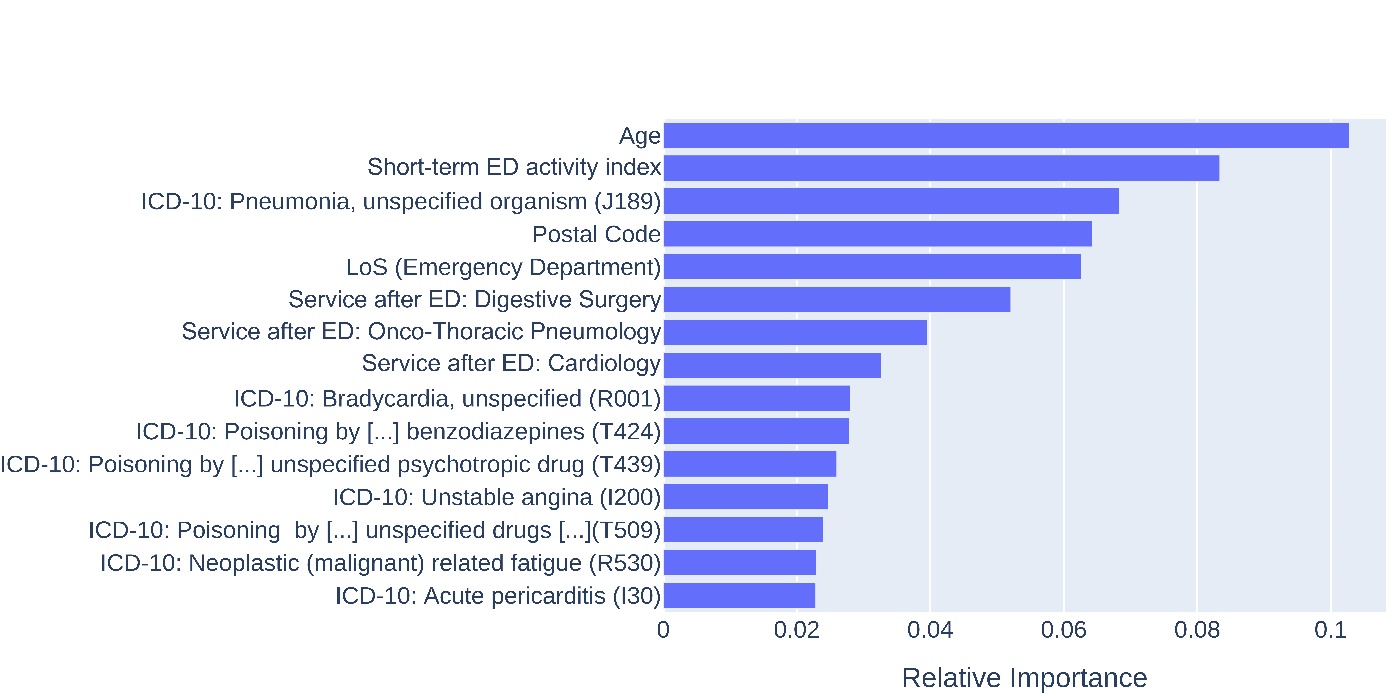
**

**Figure S5.** Feature importance for the structured data model in intensive care unit (ICU) patients to predict hospital length of stay (LOS)
