# Supplementary material for: The prediction of hospital length of stay using unstructured data
Source: BMC Med Inform Decis Mak. 2021 Dec 18;21:351. doi: 10.1186/s12911-021-01722-4 (PMC8684269; doi:10.1186/s12911-021-01722-4)
Supplement: Supplementary file 2 — Additional file 2. Data formats used for modeling. [file 12911_2021_1722_MOESM2_ESM.docx]

**Appendix 2**

Data formats used for modelling (model matrix)

| **Structured data model input: fully anonymized excerpt data** | | |  |  |  |  |  |  |  |  |  |  |  |  |  |  |
| --- | --- | --- | --- | --- | --- | --- | --- | --- | --- | --- | --- | --- | --- | --- | --- | --- |
| **Age** | **Gender** | **Postal Code** | **LoS in the ED** | **Recent prior visit** | **Short-term ED activity index** | **Service after ED: Short Stay Emergency Ward** | **Service after ED: Cardiology** | **Service after ED: Digestive Surgery** | **...** | **Service after ED: Rheumatology** | **CCMU Code** | **GEMSA CODE** | **ICD-10: A090** | **ICD-10: A099** | **...** | **ICD-10: A150** |
| 62.1 | 0 | XXXXX | 0.6 | 1 | 0 | 1 | 0 | 0 | … | 0 | 1 | 4 | 0 | 0 | … | 0 |
| 53.2 | 1 | XXXXX | 0.1 | 0 | 0 | 0 | 0 | 1 | … | 0 | 2 | 2 | 0 | 1 | … | 0 |
| 44.3 | 0 | XXXXX | 0.9 | 0 | 1 | 0 | 0 | 0 | … | 0 | 4 | 1 | 0 | 0 | … | 0 |
| … | … | … | … | … | … | … | … | … | … | … | … | … | … | … | … | … |
|  |  |  |  |  |  |  |  |  |  |  |  |  |  |  |  |  |
| **Unstructured data model input: fully anonymized excerpt data** | | |  |  |  |  |  |  |  |  |  |  |  |  |  |  |
| **Age** | **Gender** | **Postal Code** | **LoS in the ED** | **Recent prior visit** | **Short-term ED activity index** | **Service after ED: Short Stay Emergency Ward** | **Service after ED: Cardiology** | **Service after ED: Digestive Surgery** | **...** | **Service after ED: Rheumatology** | **UMLS: Cutaneous abscess** | **UMLS: Respiratory Alkalosis** | **....** | **UMLS : Pulmonary Tuberculosis** |  |  |
| 62.1 | 0 | XXXXX | 0.6 | 1 | 0 | 1 | 0 | 0 | … | 0 | 1 | 1 | … | 0 |  |  |
| 53.2 | 1 | XXXXX | 0.1 | 0 | 0 | 0 | 0 | 1 | … | 0 | 0 | 1 | … | 0 |  |  |
| 44.3 | 0 | XXXXX | 0.9 | 0 | 1 | 0 | 0 | 0 | … | 0 | 0 | 0 | … | 0 |  |  |
| … | … | … | … | … | … | … | … | … | … | … | … | … | … | … |  |  |

LoS: Length of Stay; ED: Emergency Department; GEMSA: Multicentric classification of non-programmed care patients; ICD-10: International Classification of Diseases, 10th Edition; UMLS: Unified Medical Language System
